# Outcomes and Toxicities of Nonmedullary Thyroid Tumors Treated with Proton Beam Radiation Therapy

**DOI:** 10.14338/IJPT-22-00005.1

**Published:** 2022-07-15

**Authors:** Irini Youssef, Jennifer Yoon, Nader Mohamed, Kaveh Zakeri, Robert H. Press, Yao Yu, Jung Julie Kang, Richard J. Wong, R. Michael Tuttle, Ashok Shaha, Eric Sherman, Nancy Y. Lee

**Affiliations:** 1 Department of Radiation Oncology, SUNY Downstate Health Sciences University, Brooklyn, NY, USA; 2 Department of Radiation Oncology, Rutgers Cancer Institute of New Jersey, New Brunswick, NJ, USA; 3 Department of Radiation Oncology, Memorial Sloan Kettering Cancer Center, New York, NY, USA; 4 New York Proton Center, New York, NY, USA; 5 Department of Head and Neck Surgery, Memorial Sloan Kettering Cancer Center, New York, NY, USA; 6 Department of Endocrinology, Memorial Sloan Kettering Cancer Center, New York, NY, USA; 7 Department of Medical Oncology, Memorial Sloan Kettering Cancer Center, New York, NY, USA

**Keywords:** protons, nonmedullary, thyroid, toxicity, Bragg peak

## Abstract

**Purpose:**

Proton therapy is an emerging therapy for several malignancies owing to its favorable therapeutic ratio. There are very limited data on the use of proton therapy in the management of thyroid carcinoma. Our objective was to review the safety, feasibility, and outcomes of proton therapy for patients with thyroid cancer treated to the head and neck.

**Methods:**

From our institution's proton database from 2012 to 2021, we identified 22 patients with thyroid cancer treated with proton beam therapy. We evaluated outcomes and toxicities.

**Results:**

Median follow-up was 26 months. Of the 22 patients, 50% were female. The mean age was 65 years. Three patients had anaplastic cancer; 13, papillary carcinoma; 2, follicular carcinoma; and 2, poorly differentiated carcinoma. Forty-six percent had T4 disease. Primary targets were the central neck compartment, level VI, and upper mediastinum. Radiation dose was 60 GyRBE adjuvantly, and 70 GyRBE for gross disease (range, 6000-7600 GyRBE). Eight patients underwent upfront adjuvant radiation, and 3 received definitive radiation for unresectable disease upfront. Eleven patients received either salvage or palliative radiation. Fifty-nine percent of patients had extrathyroidal extension, and 64% of patients had gross disease in the neck before treatment. Fifty percent of patients had metastatic disease before treatment. Sixteen patients received concurrent chemotherapy, 63% of these patients received doxorubicin. For all patients, 1-year local regional recurrence (LRR) was 0%, and overall survival (OS) was 90%. Acute grade 3+ toxicities occurred in 27% of patients, the most frequent being dermatitis (27%). Three patients required a percutaneous endoscopic gastrostomy tube after radiation therapy (RT), 2 owing to progression. There were no grade 4+ toxicities.

**Conclusions:**

Proton therapy for thyroid cancer appears feasible and effective with minimal toxicities. Prospective studies comparing proton therapy with intensity-modulated RT, to evaluate the clinical efficacy of using proton therapy to reduce toxicities in patients undergoing radiation for thyroid cancer, are warranted.

## Introduction

Thyroid cancer represents about 3% of all new cancer cases diagnosed in the United States each year [1]. A total of 44,280 cases were diagnosed in 2021[1], and the incidence continues to rise owing to improved detection [2]. Despite being the mainstay of therapy, complete surgical resections are often difficult owing to the proximity of the thyroid to critical anatomic structures, and resections are more complicated when these critical structures are themselves invaded by tumor. Additionally, when disease recurs, re-resections are associated with significant challenge and morbidity [3]. Thus, local regional disease control is an important endpoint when examining outcomes of thyroid cancer treatment.

External beam radiation has been demonstrated retrospectively to improve local regional control [4–6]. However, photon beam radiation is still associated with significant toxicity due to the high doses used near critical structures in the head and neck. The use of proton beam radiation has recently gained substantial momentum owing to improved accessibility to the technology and interests in its advantage of better tissue sparing when compared to traditional photon radiation [7].

Proton beam radiation is being used increasingly for head and neck cancers. Very little literature exists on the formal use of protons in the management of thyroid cancer. We report our institutional experience of using protons in the management of thyroid cancer.

## Patients and Methods

This study was approved by the Memorial Sloan Kettering Cancer Center (MSKCC) and New York Proton Center (NYPC) Institutional Review Board and a waiver of informed consent was granted because of the retrospective nature of the study. We retrospectively reviewed all cases of patients treated between 2012 and October 2021 for thyroid cancer, using proton radiation at MSKCC. This included patients receiving definitive and palliative radiation. Twenty-two patients with nonmedullary thyroid cancer were included in the final analysis. At the time of diagnosis or recurrence, a history and physical examination were completed for all patients by an endocrinologist, head and neck surgeon, medical oncologist, and radiation oncologist. Staging computed tomography and magnetic resonance imaging or positron emission tomography imaging were obtained if necessary for management.

Intensity-modulated proton therapy (IMPT) was generally offered as an alternative to intensity-modulated radiation therapy (IMRT) off trial or was necessitated for large tumors when IMRT could not be safely delivered. The reasons for patients not receiving IMPT included patient's preference for IMRT as the standard of care; insurance denial of IMPT; and logistic issues for daily visits to the proton treatment centers, which are located at different campuses from the MSKCC campus. The study included patients treated at both Procure (Somerset, New Jersey) and NYPC (New York, New York). Proton therapy was delivered by using a Proteus 235 system (Ion Beam Applications, Louvain-la-Neuve, Belgium) or a Varian ProBeam system (Varian Medical Systems, Palo Alto, California) at NYPC, treating with either uniform scanning or pencil beam scanning (PBS). Pencil beam scanning using 3 to 5 fields was the preferred modality once available. Most patients undergoing PBS proton therapy were planned with single-field uniform dose (SFUD), and multi-field optimization (MFO) was reserved for more complicated cases (typically larger or more locally advanced tumors) to maximize conformality and reduce dose to nearby organs at risk. In this article, we use the term *IMPT* to refer to any PBS plan (both SFUD and MFO). IMPT treatments began in December 2015. For both uniform scanning and PBS plans, setup variations were accounted for by compensator smear or simulated isocenter shift variations of between 3 and 5 mm, respectively. The clinical target volume was expanded to a planning treatment volume typically 3mm to account for intrafraction patient motion and interfraction setup error. Robustness tests, including range uncertainty of ±3.5%, were performed to ensure that clinical target volume V95 > 95%. A generic relative biological effectiveness of 1.1 was used for dose evaluation. All patients were aligned by using orthogonal radiographs on a 6-degrees-of-freedom couch [8]. At NYPC, all patients are treated with PBS protons.

The radiation technique has been previously described [3]. Briefly, we treated various clinical target volumes as follows: low-risk areas including elective lymph node volumes to 54 GyRBE; high-risk areas including the operative or tumor bed, operative thyroid gland volume, tracheoesophageal grooves, and central nodal compartment to 60 GyRBE; close or microscopically positive margins to 66 GyRBE; and areas of gross disease to 70 GyRBE. Inclusion of elective lateral neck nodal volumes was at the discretion of the treating physician. The gross tumor volume was defined as the gross extent of tumor visible by imaging studies and clinical examination. Patients not candidates for definitive management were treated with the QUAD-shot regimen. This regimen is defined as 3.7 GyRBE delivered in 4 fractions given twice daily at least 6 hours apart on 2 consecutive days. If the patient tolerates this regimen and the tumor does not progress, it can be repeated every 4 weeks. The clinical target volume was expanded to a planning target volume typically 3 mm to account for intrafraction patient motion and interfraction setup error. All patients were immobilized in the treatment position by using a 3- or 5-point Aquaplast mask.

### Statistical Analysis

Patient, tumor, and treatment characteristics were recorded and analyzed with descriptive statistics. Adverse events (AEs) occurring within 180 days after radiation treatment completion were considered acute, while those occurring after the 180-day period were considered late AEs, and were graded and recorded according to the Common Terminology Criteria for Adverse Events (CTCAE) version 5.0 (US National Cancer Institute, Bethesda, Maryland). Radiation treatment records and oncologic outcomes were manually abstracted from electronic medical records, using a uniform data abstraction form. Local or regional failures were confirmed by both imaging studies and tissue biopsies. Local regional recurrence (LRR), local regional recurrence–free survival (LRR-FS), progression-free survival (PFS), and overall survival (OS) were calculated from the start of radiation therapy (RT) until the occurrence of events or censoring. Local regional recurrence was defined as progression or recurrence of disease within the neck. Progression-free survival was calculated as survival without local, regional, or distant metastatic progression. LRR-FS was defined as patients alive, without progression in the head and neck. All analyses were performed with SPSS 2021, version 28.0 (IBM, Armonk, New York).

Data regarding patient sex, age, and baseline performance status were collected for all 22 patients. T, N, and M staging, as well as presence of gross or metastatic disease at time of radiation, was collected for all patients. The reported M (distant metastasis) category was recorded at the time of IMRT start. For patients who underwent surgical resection, the number of resections and date of last surgery were collected, in addition to pathologic characteristics including histology, extrathyroidal extension, extranodal extension, perineural invasion, and presence of microscopic or macroscopic disease. Unresectable disease category includes patients with upfront unresectable disease and those with unresectable recurrent disease. Data regarding receipt of radioactive iodine were abstracted if available, in addition to the number of radioactive iodine sessions received. Patients treated within 120 days of surgery were considered to be in the adjuvant group, and those treated >120 days of surgery were considered to be in the salvage group. Fisher exact test was used to compare toxicities between patients receiving concurrent chemotherapy versus radiation alone. Kaplan-Meier survival analysis was used to calculate LRR and OS.

## Results

**Figure 1.**
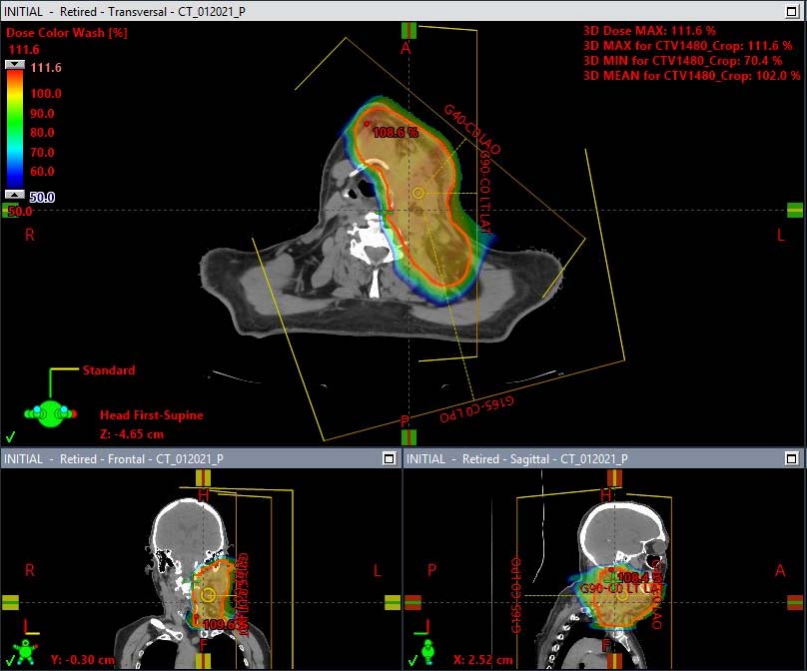
This is a patient with anaplastic thyroid cancer treated with thyroidectomy and radioactive iodine, presenting with recurrence invading into the neck musculature, encasing the left internal carotid artery, compressing the internal jugular vein, with extension into floor of mouth and left prevertebral space. The patient received 2 cycles of QUAD shot radiation to a total dose of 2960 GyRBE, as well as concurrent chemotherapy with doxorubicin and dacarbazine. At last follow-up 12 months later, the disease has not progressed locally or systemically.

Median follow-up since completion of IMPT for all 22 patients was 26 months (range, 2-74 months). Median age at treatment was 65 years, and 50% of patients were female. Twenty of 22 patients (91%) had a Karnofsky performance status ≥80. Histologic types included papillary (63.6), follicular (9.1%), and poorly differentiated (13.6%) carcinoma, with 3 patients having anaplastic cancer (13.6%). Most patients had at least 1 biopsy-proven recurrent disease (73%, N = 16). Median number of resections before proton radiation was 2 (range, 0-4). Fifty percent of patients (N = 11) had metastatic disease at radiation. Sixteen patients received concurrent chemotherapy, 10 (63%) of whom received doxorubicin. Patient and tumor characteristics are presented in **Table 1**. An example case is demonstrated in **Figure 1**.

**Table 1.** Baseline patient and treatment characteristics.

**Patient or treatment characteristic**	**Number (%)**
Age, y	
Mean (IQR)	64.95 (53-76)
≤45 y, n (%)	1 (4.5)
>45 y, n (%)	21 (95.5)
Karnofsky performance status, median (range)	90 (60-100)
No. of operations, n (%)	
0	3 (13.6)
1	7 (30.4)
2	6 (26.1)
3	4 (17.4)
4	2 (8.7 )
Follow-up, median (range), mo	26 (2-74)
Tumor stage, n (%)	
T1	1 (4.5)
T2	3(13.6)
T3	6 (27.3)
T4	12( 54.5)
Nodal stage, n (%)	
N0	4 (18.2)
N1	18 (81.8)
Prognostic stage (AJCC 8th edition [9]), n (%)	
1	3 (13.6)
2	8 (36.4)
3	3 (13.6)
4A	1 (4.5)
4B	7 (31.8)
Gross disease present at radiation, n (%)	
No	8 (36)
Yes	14 (64)
Extrathyroidal extension, n (%)	13 (59)
Extranodal extension, n (%)	6 (27)
R1R2 resection, n (%)	9 (41)
Perineural or gross nerve invasion, n (%)	6 (27)
No. of recurrences before proton therapy, n (%)	
0	6 (27.3)
1	8 (36.4 )
2	2 (9.1)
3	5 (22.7)
4	0 (0)
5	1 (4.5)
Radioactive iodine, n (%)	N = 14
1	9 (64.3)
2	2 (14.3)
3	1 (7.1)
4	2(14.3)
Concurrent chemotherapy, n (%)	
No chemotherapy	6 (27.3)
Carboplatin/paclitaxel	3 (13.6)
Doxorubicin	10 (45.5)
Paclitaxel	2 (9.1)
Doxorubicin/dacarbazine	1 (4.5)
Radiation dose, median (IQR), GyRBE	70 (60-70)
Definitive radiation (unresectable upfront), n (%)	3 (14)
Immediate adjuvant radiation, n (%)	8 (36)
Surgeries before any radiation, n (%)	
0	3 (13.6)
1	7 (31.8)
2	6 (27.3)
3	4 (18.2)
≥4	2 (9.1)
Salvage/palliative radiation, n (%)	11 (50)
QUAD regimen, n (%)	4 (18)
No. of QUAD cycles, n (%)	
1	1 (25)
2	1 (25)
3	0 (0)
4	2 (50)

**Abbreviations:** IQR, interquartile range; AJCC, American Joint Committee on Cancer.

Note: QUAD regimen: 7 GyRBE delivered in 4 fractions given twice daily at least 6 hours apart on 2 consecutive days.

### Oncologic Outcomes

**Figure 2.**
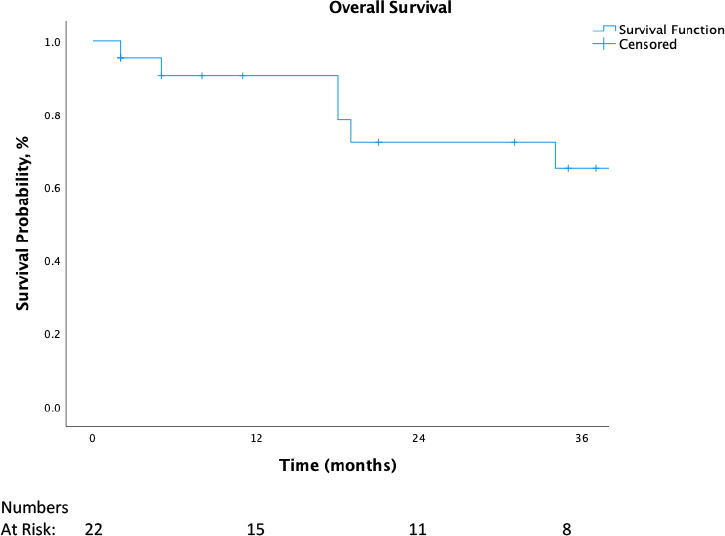
Overall survival of all patients.

**Figure 3.**
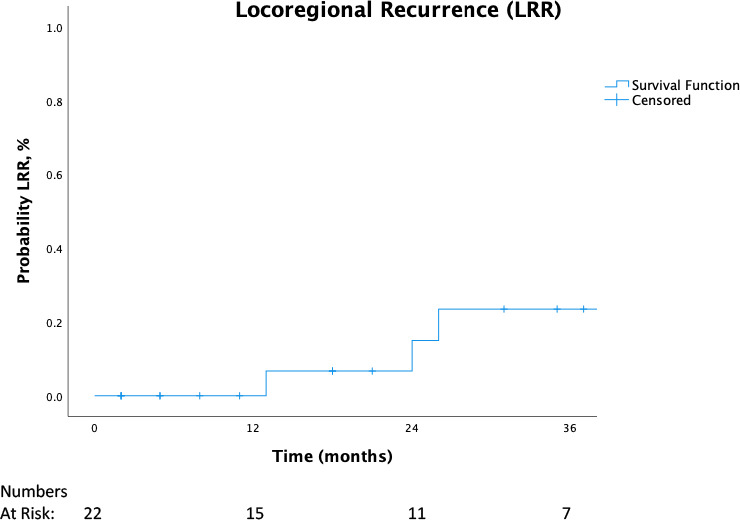
Local regional recurrence for all patients.

For all patients, OS at 12 and 24 months was 90% and 72%, respectively. LRR at 12 months was 0%, and 16% at 24 months. Overall survival and LRR for all patients are shown in **Figures 2** and **3**, respectively. Of patients treated with at least 59 GyRBE, OS at 12 months was 94%, and cumulative LRR, 0%. For all patients, LRR-FS at 12 months was 80% for those not receiving concurrent chemotherapy versus 94% for those receiving concurrent chemotherapy. Patients treated with at least 59 GyRBE who also received concurrent chemotherapy had no LRRs at 12 months.

Excluding patients with metastatic disease before radiation (N = 11), OS at 12 months was 100% (median, 21 months); LRR, 0%; and PFS, 81%. Among all patients treated with adjuvant proton radiation (N = 8), 50% of whom had metastatic disease at time of radiation, LRR at 12 months was 0% and 17% at 18 months. Among all patients treated for unresectable disease, 7 of whom also had metastatic disease at time of radiation, LRR-FS and OS at 12 months were both 84%. Four patients received QUAD shot radiation, all owing to extensive local disease and extensive metastatic disease with poor prognosis. Three of these patients had 2 prior resections before requiring palliative radiation. One patient had unresectable disease at the time of initial diagnosis. When excluding patients who received QUAD shot radiation, the LRR at 12, 24, and 48 months was 0%, 16%, and 16%, respectively.

### Toxicity Outcomes

Acute grade 3 toxicities occurred in 27% of patients. One patient experienced grade 3 dysphagia requiring percutaneous endoscopic gastrostomy (PEG) tube placement within 60 days of RT; 6 patients experienced grade 3 dermatitis; and 1, grade 3 mucositis. No patients experienced grade 4 or greater acute toxicity. Acute toxicities grade 2 or greater are reported in **Table 2**. The use of concurrent chemotherapy was not significantly associated with greater toxicity for any acute toxicity when comparing grade 1 versus grade 2 or greater toxicities (**Table 3**).

**Table 2.** Acute and chronic toxicities.

**Toxicity and grade**	**Acute, n (%)**	**Chronic, n (%)**
Odynophagia or oral pain		
0	7 (31.8)	21 (95.5)
1	5 (22.7)	1 (4.5)
2	10 (45.5)	NA
3+	NA	NA
Dysphagia		
0	5 (22.7)	13 (59.1)
1	4 (18.2)	6 (27.3)
2	12 (54.55)	3 (13.6)
3+	1 (4.55)	0 (0)
Fatigue		
0	12 (54.6)	18 (81.8)
1	7 (31.8)	2 (9.1)
2	3 (13.6)	2 (9.1)
3+	NA	NA
Xerostomia		
0	12 (54.5)	16 (72.7)
1	5 (22.7)	5 (22.7)
2	5 (22.7)	1 (4.5)
3+	NA	NA
Dysgeusia		
0	17 (77.3)	21 (95.5)
1	1 (4.5)	1 (4.5)
2	4 (18.2)	NA
3+	NA	NA
Dermatitis		
0	5 (22.7)	20 (91)
1	2 (9.1)	2 (9)
2	9 (40.9)	NA
3+	6 (27.3)	NA
Mucositis		
0	13 (59.1)	NA
1	2 (9.1)	NA
2	6 (27.3)	NA
3+	1 (4.5)	NA
Hoarseness		
0	10 (45.5)	16 (73)
1	8 (36.4)	6 (27)
2	4 (18.2)	NA
3+	NA	NA
Nausea		
0	19 (86)	NA
1	3 (14)	NA
2	NA	NA
3+	NA	NA

**Abbreviation:** NA, not available.

**Table 3.** Fisher exact test analysis of acute toxicity by treatment.

**Acute toxicity and grade**	**Radiation alone, n (%), N = 6**	**Concurrent chemotherapy, n (%), N = 16**	***P*** **value**
Odynophagia or oral pain			0.42
0–1	4 (67)	8 (50)	
2+	2 (33)	8 (50)	
Dysphagia			0.48
0–1	3 (50)	6 (37.5)	
2+	3 (50)	10 (62.5)	
Fatigue			0.64
0–1	5 (83)	14 (87.5)	
2+	1 (17)	2 (12.5)	
Xerostomia			0.58
0–1	5 (83)	12 (75)	
2+	1 (17)	4 (25)	
Dysgeusia			0.25
0–1	6 (100)	12 (75)	
2+	NA	4 (25)	
Dermatitis			0.35
0–1	1 (17)	6 (37.5)	
2+	5 (83)	10 (62.5)	
Mucositis			0.35
0–1	5 (83)	10 (62.5)	
2+	1 (17)	6 (37.5)	
Hoarseness			0.25
0–1	6 (100)	12 (75)	
2+	NA	4 (25)	
Nausea			0.25
0–1	6 (100)	16 (100)	
2+	NA	NA	

**Abbreviation:** NA, not available

Nine patients experienced late grade 1-2 dysphagia. One patient underwent PEG tube placement 90 days after radiation completion owing to dysphagia. This patient received 76 GyRBE. Two patients received PEG tubes more than 12 months after completion of RT, owing to disease progression. Three patients experienced esophageal stenosis requiring balloon dilatation. All of these patients received at least 70 GyRBE to the primary tumor volume, 1 receiving 76 GyRBE. One patient with grade 1 lymphedema was managed with physical therapy and supportive care. Five patients experienced late fibrosis, and 4 patients experienced trismus. Additional late toxicities are reported in **Table 2**.

## Discussion

The use of photon beam radiation has become an integral component in management of head and neck malignancies. While the development of intensity-modulated radiation therapy has had a great impact on reductions in toxicity through its conformity, the radiation of normal tissue remains inevitable owing to the physical properties of photon beams [10]. This can lead to unnecessarily debilitating acute and chronic toxicities. The unique physical properties of proton therapy, namely its ability to concentrate most of its biological impact to the “Bragg peak”, allows high doses to be delivered to the designated tumor volume, while the minimal distal falloff allows sparing of normal tissue [10]. Increasing availability and access to proton beam radiation has made it a promising option.

Proton radiation has been shown to have superior normal tissue sparing with less treatment-related toxic effects, compared with photon radiation, in the treatment of several head and neck cancer subtypes, such as oropharyngeal cancer [11], salivary gland tumors [12], and sinonasal cancer [13]. A recent retrospective analysis by Li et al [14] showed that IMPT is associated with lower likelihood of developing grade 2 or higher acute AEs, compared with IMRT, in patients treated for nonmetastatic nasopharyngeal carcinoma. Only 1 case (3.8%) of a chronic grade 3 or higher AE occurred in the IMPT group compared with 8 cases (16.3%) in the IMRT group. Additionally, there was no statistically significant difference in LRR between the proton and photon groups. Aljabab et al [15] also reported a favorable toxicity profile without any local regional or marginal recurrences in 46 patients with stage III-IV oropharyngeal carcinoma treated with proton radiation.

The use of radiation in the management of thyroid cancer remains less well defined given the lack of randomized prospective data. For patients with radioactive iodine refractory disease, radiation may play an important role in improving local regional control. The role of proton beam radiation in the management of thyroid cancer is even more equivocal. Our institution's experience demonstrates that proton radiation is a feasible option associated with minimal acute and late toxicity.

Very little literature exists exploring the use of photon beam radiation for thyroid cancer. We previously reported on the use of definitive intent photon-based radiation therapy in 88 patients with nonanaplastic, nonmedullary thyroid cancer, with or without concurrent chemotherapy. Patients receiving concurrent chemotherapy (CC)-IMRT had higher local progression-free survival (LPFS) than with IMRT alone (CC-IMRT 85.8% versus IMRT 68.8%, *P* = 0.036). Additionally, grade 3+ acute toxicities occurred in 23.9% of patients, the most frequent being dermatitis (18.2%) and mucositis (9.1%). In all 17.1% of patients required a PEG tube during or shortly after completion of RT, with 10.1% of patients needing a PEG tube more than 12 months after therapy [3]. A study by Yu et al [16] reported on 14 patients with differentiated thyroid cancer who received IMPT. This study reported a 100% survival rate at 8 months follow-up, with acute grade 3 toxicity limited to 1 patient, and only 2 patients having late grade 3 toxicity. There were no grade 4 or 5 toxicities. Our study demonstrated that proton beam radiation offers comparable outcomes. Only 1 patient required PEG tube placement during or within 60 days of RT, 6 had grade 3 dermatitis, and 1 had grade 3 mucositis. These toxicities were not statistically significantly higher in patients who also received concurrent chemotherapy, while still maintaining an LRR-FS rate at 12 months of 100% in patients receiving at least 59 GyRBE.

One experience from Beijing of 405 patients reported an improvement in 5-year survival in patients with incomplete surgeries using external-beam radiation therapy (71% versus 33%) [17]. In our study, of all patients with gross disease in the neck before RT, without disease elsewhere, only 1 had died at last follow-up. This patient was treated with QUAD regimen. In their pooled analysis, Fussey et al [18] demonstrated a recurrence rate of 8%. In our study, across all patients, including those receiving definitive and palliative radiation, the cumulative LRR rate was 0% at 12 months, 15% at 24 months, 24% at 36 months, and 43% at 4 years. For patients without metastatic disease at time of radiation, cumulative incidence of LRR rate was 0% at 24 months and 20% at last follow-up.

Currently, there is no set standard for allocation of patients to proton therapy. In our institution, patients are offered proton therapy in the settings of active trials, as an alternative to IMRT when it could not be safely delivered, in the setting of re-irradiation, or patient preference. Additional normal tissue control probability and toxicity risk reduction models have been described [19, 20]. In a scoping review of patient selection models for proton therapy in head and neck cancers, Zientara et al [21] reported a few methods including but not limited to informed decision-making, cost effectiveness, normal tissue complication probability comparisons between plans, prediction software comparing doses to the tumor volume and organs at risk, and multidisciplinary team consensus. In addition to these selection models, patients should optimally be enrolled into clinical trials comparing protons to photons.

This study has its limitations. Firstly, it is a retrospective study, with a small cohort of patients. However, it is the largest study published to date evaluating the role of proton beam radiation in the management of fully resected, locally confined, or metastatic thyroid cancer. We are also aware that this study combines different thyroid histologic types with different behaviors. Another limitation is the lack of a comparison group. However, this is a study on both oncologic outcomes and toxicity data of patients treated at a high-volume academic tertiary care center. Altogether, our findings demonstrate that proton beam radiation is both safe and feasible in the management of patients with thyroid cancer, including those with metastatic, gross, and radioactive iodine–refractory disease, without compromising oncologic outcomes.

## Conclusion

The use of proton therapy in the management of nonmedullary thyroid cancers appears to be safe, feasible, and effective. However, prospective studies are needed to compare photon therapy to proton therapy in order to evaluate the clinical efficacy of using proton radiation's “Bragg peak” properties to reduce toxicities in patients needing radiation therapy.
